# A novel small-form NEDD4 regulates cell invasiveness and apoptosis to promote tumor metastasis

**DOI:** 10.18632/oncotarget.3322

**Published:** 2015-03-20

**Authors:** Chia-Jung Liao, Hsiang-Cheng Chi, Chung-Ying Tsai, Chi-De Chen, Sheng-Ming Wu, Yi-Hsin Tseng, Yang-Hsiang Lin, I-Hsiao Chung, Ching-Ying Chen, Syuan-Ling Lin, Shiu-Feng Huang, Ya-Hui Huang, Kwang-Huei Lin

**Affiliations:** ^1^ Department of Biochemistry, Chang-Gung University, Taoyuan, Taiwan 333, Republic of China; ^2^ Chang Gung Molecular Medicine Research Center, Chang-Gung University, Taoyuan, Taiwan 333, Republic of China; ^3^ Institute of Molecular and Genomic Medicine, National Health Research Institutes, Zhunan, Miaoli, Taiwan 350, Republic of China; ^4^ Medical Research Center, Chang Gung Memorial Hospital, Taoyuan, Taiwan 333, Republic of China

**Keywords:** Hepatoma, prognosis, sNEDD4, metastasis, apoptosis

## Abstract

Despite numerous investigations on metastasis, the determinants of metastatic processes remain unclear. We aimed to identify the metastasis-associated genes in hepatocellular carcinoma (HCC). Potent metastatic SK-hep-1 (SK) cells, designated ‘SKM’, were generated using Transwell assay followed by selection in a mouse model. Genes expressed differentially in SKM and SK cells were identified via microarray analyses. A small form of Neural precursor cell-expressed developmentally downregulated 4 (sNEDD4) was identified to be overexpressed in SKM cells, which was confirmed as a novel transcript using liquid chromatography-mass spectrometry. In clinical specimens, sNEDD4 was significantly overexpressed in tumors and serves as a poor prognostic factor for male patients with HCC (*P* = 0.035). Upon subcutaneous introduction of sNEDD4-overexpressing SK cells into flanks of nude mice, tumors grew faster than those of the control group. Furthermore, sNEDD4-mediated promotion of tumor metastasis was demonstrated in the orthotopic mouse model. Overexpression of sNEDD4 increased the invasive ability of SK cells through upregulation of matrix metalloproteinase 9 and inhibited serum deprivation-induced apoptosis via upregulation of myeloid cell leukemia 1. In conclusion, sNEDD4 is a novel metastasis-associated gene, which prevents apoptosis under nutrient restriction conditions. The present findings clearly support the prognostic potential of sNEDD4 for HCC.

## INTRODUCTION

Metastasis, the dissemination of cancer cells from the primary tumor and their colonization at a secondary site, is responsible for 90% of cancer-associated deaths. It is a complex process with multiple steps comprising local invasion, intravasation, survival in the circulation, extravasation, and growth of metastases. Despite significant improvements in our knowledge on metastasis, no effective treatments are available at present. Consequently, to optimize therapeutic options, there is an urgent medical need to identify key molecules for early detection, prevention, prognosis, and antagonizing progression of metastasis.

Several *in vivo* mouse models, such as experimental and spontaneous metastasis models, have been used [1]. Owing to the biological complexity of metastasis, no single model is sufficient to answer all our queries, and therefore selection of an optimal model to clarify each biological process is necessary. In the current study, an experimental metastasis model was utilized for selection of cells with highly metastatic properties. DNA microarray analyses comparing parental cells with selected highly metastatic cells were subsequently performed to identify metastasis-associated genes.

## RESULTS

### Generation of potent metastatic SK-Hep-1 (SK) cell lines, SKT and SKM

**Figure 1:**
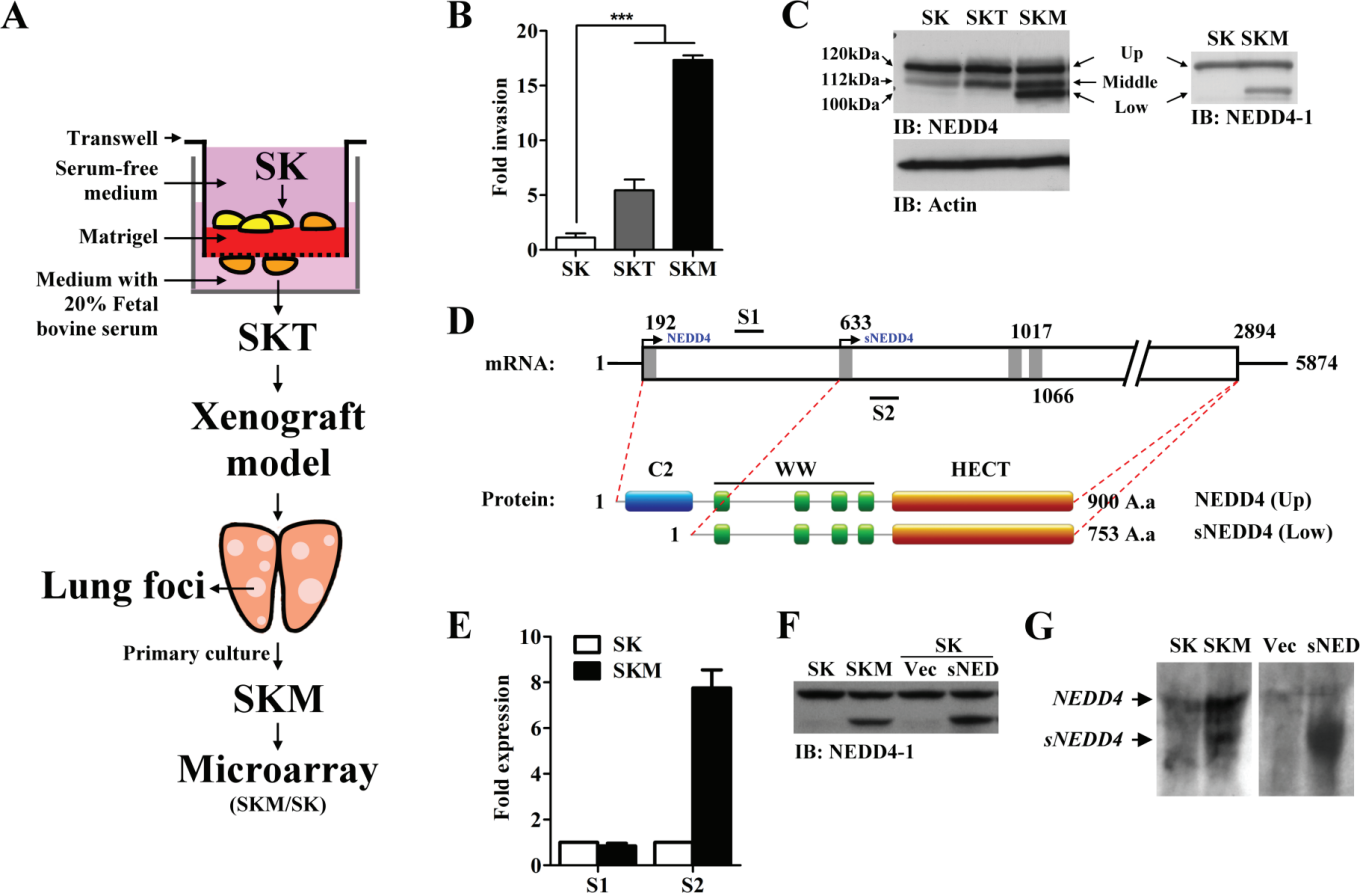
Generation of potent metastatic cell lines, SKT and SKM, and identifying metastatic-associated gene, sNEDD4 **(A)** Schematic diagram of the experimental procedure. **(B)** The invasive abilities of SK, SKT, and SKM cells were determined with the Transwell invasion assay. **(C)** Western blot analysis was used to determined NEDD4 protein expression of SK, SKT, and SKM cells. Three (left; upper, middle, and lower) and two (right; upper and lower) major bands were detected using NEDD4 and NEDD4–1 antibodies in SKM cells, respectively. **(D)** Schematic diagram of *NEDD4* mRNA (Upper). The open box represents the coding region of *NEDD4*. Classic and predictive ATG sites are labeled with gray bars. The lower schematic diagram represents NEDD4 and sNEDD4 protein structures. **(E)** NEDD4 mRNA levels were determined via qRT-PCR with primers amplified from two different segments, S1 and S2 (mark in panel D). **(F)** NEDD4 and sNEDD4 protein levels of SK, SKM and SK cells transiently transfected with empty vector (Vec) or sNEDD4 expression plasmid (sNED). **(G)** *sNEDD4* was detected using Northern blot. SK cells transfected with sNEDD4 expressing plasmid (sNED) serve as a positive control. IB, immunobloting; S1, 366–478 nucleotides; S2, 649–751 nucleotides; ***P < 0.001.

The *in vitro* Transwell assay and *in vivo* mouse model were employed to select cells with potent metastatic properties (SKT and SKM cells), with the aim of identifying metastasis-regulating genes. Experimental procedures are summarized in Figure 1A. The invasive properties of SK, SKT and SKM cells were determined using the Transwell invasion assay. Our results showed that the invasion abilities of SKT and SKM cells are significantly increased, compared to that of SK parental cells (Figure 1B). However, analysis of proliferation activity revealed no significant differences between the cell lines (data not shown). The potent metastatic cells were successfully generated.

### Identification of metastasis-associated genes

Differentially expressed genes in SKM cells, compared to SK cells, were identified using DNA microarray. cDNA and Affymetrix oligo microarrays performed in parallel led to the identification of 181 (84 upregulated and 97 downregulated) and 199 (76 upregulatied and 123 downregulated) probes with differential expression greater than 1.5-fold, respectively (data not shown). We detected 24 differentially expressed genes simultaneously from both microarray platforms (Table 1). Expression levels of several genes were verified using real-time PCR (qRT-PCR; Supplementary Figure S1). Among these, neural precursor cell-expressed developmentally downregulated 4 (NEDD4) was the most highly expressed in SKM cells (Table 1), and consequently selected for further study. NEDD4 belongs to a family of ubiquitin ligases (E3) characterized by protein structure similarity. Each member of this family contains an N-terminal C2 domain, 2–4 WW domains, and a HECT-type E3 ligase domain. NEDD4 is involved in diverse cellular processes, including regulation of cell trafficking, stability and signaling of membrane proteins, and virus budding [2]. Recent studies have revealed roles of NEDD4 in cancer [3]. PTEN, one of the major tumor suppressors, was recently shown to be ubiquitylated and degraded by NEDD4 [4]. In keeping with its proposed oncogenic function, NEDD4 has been shown to be overexpressed in several cancer types, including prostate and bladder cancer [4]. However the specific roles of NEDD4 in the processes of metastasis and its clinical significance in HCC are currently unknown.

**Table 1:** Metastasis associated genes

Symbol	ID	Title	Fold^#^
Upregulation			
NEDD4	Hs.1565	neural precursor cell expressed, developmentally down-regulated 4	3.01
S100A2	Hs.413843	S100 calcium binding protein A2	2.65
NT5E	Hs.153952	5′-nucleotidase, ecto (CD73)	2.59
TAGLN	Hs.410977	transgelin	2.22
CDA	Hs.72924	cytidine deaminase	2.20
PRPS1	Hs.56	phosphoribosyl pyrophosphate synthetase 1	2.16
ANPEP	Hs.1239	aminopeptidase N	1.99
CXCL1	Hs.789	chemokine (C-X-C motif) ligand 1	1.92
IL6	Hs.512234	interleukin 6	1.83
COL5A2	Hs.283393	collagen, type V, alpha 2	1.74
DNAJB6	Hs.181195	DnaJ (Hsp40) homolog, subfamily B, member 6	1.67
CAV1	Hs.74034	caveolin 1, caveolae protein, 22kDa	1.65
RAGE	Hs.104119	renal tumor antigen	1.62
SERPINE1	Hs.414795	serine proteinase inhibitor, clade E, member 1	1.60
Downregulation			
GEM	Hs.79022	GTP binding protein overexpressed in skeletal muscle	0.63
TKT	Hs.89643	transketolase	0.62
KIAA1102	Hs.156761	KIAA1102 protein	0.61
EPB41L2	Hs.440387	erythrocyte membrane protein band 4.1-like 2	0.60
PEA15	Hs.194673	phosphoprotein enriched in astrocytes 15	0.60
FHL2	Hs.8302	four and a half LIM domains 2	0.59
PFKP	Hs.498489	phosphofructokinase, platelet	0.58
SGCE	Hs.409798	sarcoglycan, epsilon	0.57
PME-1	Hs.63304	protein phosphatase methylesterase-1	0.51
OXTR	Hs.2820	oxytocin receptor	0.48

# Fold: Average of fold change of SKM/SK in cDNA and Affymetrix oligo microarrays

### NEDD4 protein is overexpressed in SKM cells

Expression patterns of NEDD4 in SK, SKT and SKM cells were evaluated. Two major immunoreactive bands (Figure 1C, upper and middle; 120 and 112 kDa, respectively) were observed in SK cells using the NEDD4 primary antibody, which recognizes both NEDD4 and NEDD4-like (NEDD4-L) proteins. Interestingly, an extra band with a smaller size of about 100 kDa (Figure 1C, low) was detected in SKM, but not SK and SKT cells. NEDD4-neutralizing peptides were used to assess the specificity of the NEDD4 primary antibody. As shown in Supplementary Figure S2A, all three immunoreactive bands were competed with NEDD4-neutralizing peptides, but not Glutathione-S transferase (GST) control peptides. The three bands were subsequently immunoprecipitated (Supplementary Figure S2B) and identified using liquid chromatography-mass spectrometry (LC-MS/MS). Overall, 17, 2, and 4 independent peptides representing NEDD4 were identified from the upper-, middle-, and lower-immunoreactive bands, respectively. Thirteen independent peptides identified from the middle-bands refer to NEDD4-L (Supplementary Table S1). Upon Western blot with the NEDD4–1 antibody, which does not recognize NEDD4-L, only the upper- and lower-immunoreactive bands were observed (Figure 1C right panel). These data further confirmed the results of LC-MS/MS.

### A novel NEDD4 transcript is overexpressed in SKM cells

The possibility of whether the short-form of NEDD4, sNEDD4, is a degradation product of NEDD4 was ruled out by treating cells with MG132 (data not shown), a proteasome inhibitor, and subsequent examination of NEDD4 transcripts. Four other NEDD4 variants (NM_198400.3, NM_001284338.1, NM_001284229.1, NM_001284340.1) have been identified to date, with longer amino acid sequences encoding proteins bigger than 120 kDa (Supplementary Figure S3). Therefore, sNEDD4 is not a product of a known transcript variants. We hypothesized that sNEDD4 is a novel transcript with an alternative downstream potential translational start site. A website tool, Promoter 2.0 Prediction Server, was used to predict the potential translational start site labeled with gray bars in Figure 1D. A^192^TG is the classical ATG site, while A^633^TG, A^1017^TG, and A^1066^TG are predictive ATG sites. Transcription of sNEDD4 from position 633 or 1017 results in a short in-frame product, and transcription from site 1066 causes frame shift reading. We designed two pairs of primers to amplify the segments upstream (Segment 1, S1; nucleotide 366–478) and downstream (Segment 2, S2; nucleotide 649–751) of the predicted A^633^TG site, respectively, and amplicons were quantified using qRT-PCR. As shown in Figure 1E, no significant differences in the expression patterns of upstream amplicons (S1) were observed in SK and SKM cells, while the downstream amplicon (S2) level was significantly increased in SKM cells. The increased level of the 649–751 amplicon is consistent with upregulation of sNEDD4 protein in SKM cells. A sNEDD4 expression plasmid (encoding nucleotides 596–2894) was generated and transfected into SK cells. sNEDD4 was successfully expressed by SK cells transfected with the expression plasmid, and the molecular weight of exogenous sNEDD4 was identical to that of endogenous sNEDD4 in SKM cells (Figure 1F). Furthermore, a smaller sNEDD4 transcript was observed in SKM but not in SK cells by Northern blot analysis (Figure 1G).

### sNEDD4 is overexpressed in human hepatocellular carcinoma (HCC)

**Figure 2:**
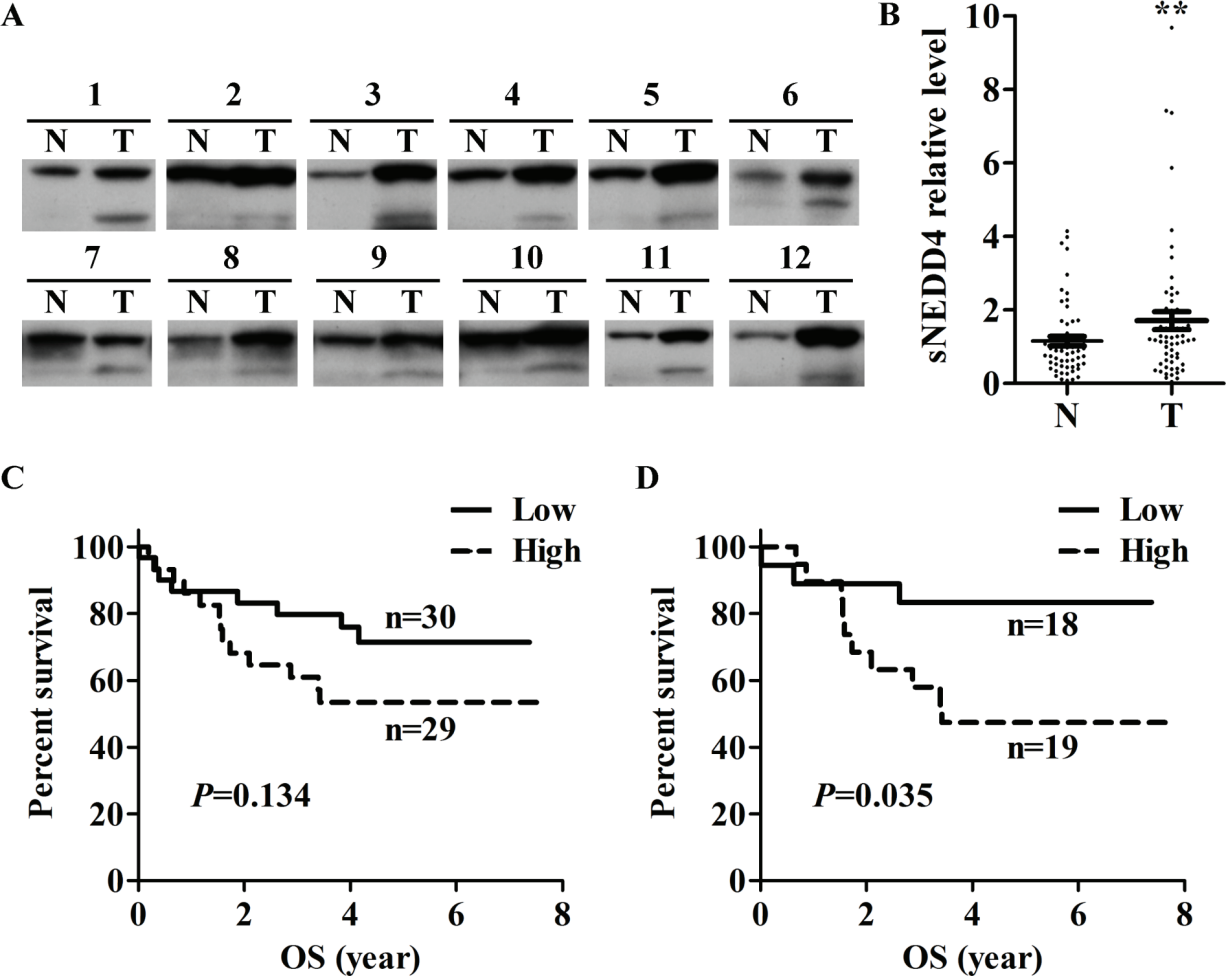
Expression of sNEDD4 in HCC sNEDD4 protein levels were verified in 70 paired adjacent normal and tumor liver tissues. **(A)** Representative results of Western blot analysis are shown. **(B)** Quantitative results of Western blot. Survival curves of low- and high- expression sNEDD4 groups of the entire study population **(C)** and male subgroup **(D)**. N, adjacent normal; T, tumor; **P < 0.01; OS, overall survival.

In order to investigate whether sNEDD4 is expressing in human livers, expression of sNEDD4 in 70 paired adjacent normal and HCC specimens was determined using Western blot analysis (Figure 2A). The relative intensities of sNEDD4 and NEDD4 were quantified, and statistical analyses performed. Expression levels of sNEDD4 and NEDD4 were significantly higher in tumor tissues than adjacent normal counterparts (Figure 2B and data not shown; *P* = 0.008 and 0.001, respectively). Tumor-to-normal tissue ratio of sNEDD4 or NEDD4 according to clinical parameters was determined. No significant differences were observed among patients with different ages, tumor sizes, grades, tumor types, vascular invasion status, stage, and cirrhosis status. However, T/N ratios of sNEDD4 were significantly different among patients with different viral status (HBV and HCV vs. NBNC; 1.99 ± 2.1 and 0.7 ± 0.49, respectively; *P* = 0.015). sNEDD4 T/N ratios of liver tissues were dichotomized according to the media ratio as low- and high-expression groups. Survival of patients with high and low sNEDD4 expression was presented as Kaplan-Meier survival curves (Figure 2C and 2D). In all study populations, patients in the high sNEDD4 expression group displayed a poorer overall survival (OS; Figure 2C) and recurrence-free survival (RFS; data not shown) trends, compared to those in the low expression group. In male populations, OS (but not RFS) of patients with high sNEDD4 expression was significantly poorer than those with low sNEDD4 expression (Figure 2D). However, no significant differences in survival were observed in female populations with different sNEDD4 expression status. Determination of the clinical significances of NEDD4 revealed no significant correlations with clinical parameters or differences in survival of patients with different NEDD4 status (data not shown).

### sNEDD4 displays more potent effect on liver cancer cell invasion than NEDD4

**Figure 3:**
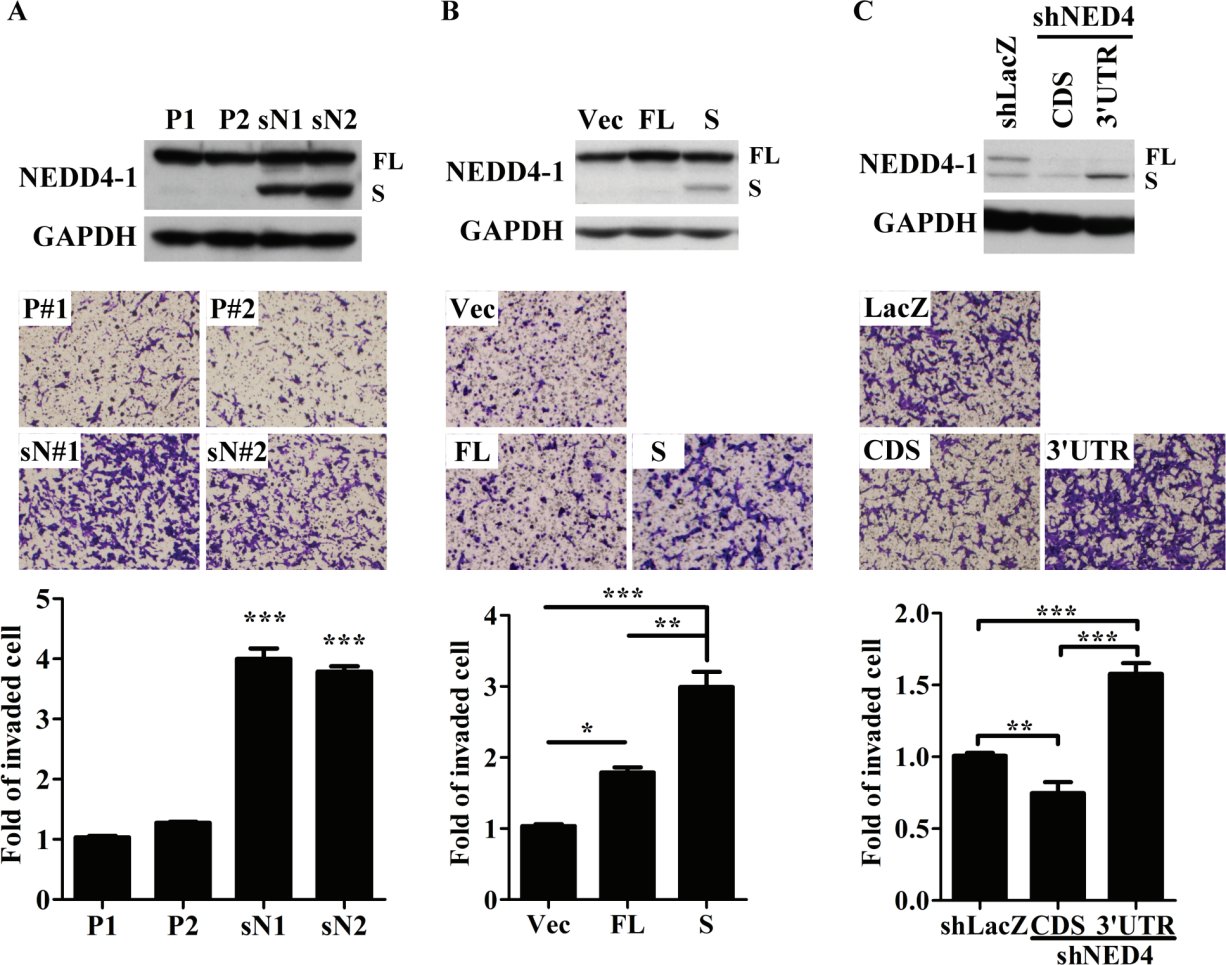
sNEDD4 promotes tumor cell invasion *in vitro* NEDD4 and sNEDD4 protein levels of cells were determined using Western blot (**A–C** upper panel). The invasive ability of cells was determined with the Transwell invasion assay (**A–C**, middle and lower panel). P1 and P2, control stable SK cell lines; sN1 and sN2, stable sNEDD4-overexpressing SK cells; Vec, empty vector control; FL, full-length NEDD4; S, sNEDD4; CDS, NEDD4 shRNA target to coding region; 3′UTR, NEDD4 shRNA target to the 3′-untranslated regions; *P < 0.05; **P < 0.01; ***P < 0.001.

To determine the effect of sNEDD4 on tumor invasion, stable control (P1 and P2) and sNEDD4-overexpressing (sN1 and sN2) SK cell lines were generated (Figure 3A, upper panel). The invasive abilities of stable cells were determined using Transwell invasion analysis (Figure 3A, middle and lower panels). Our results showed that the invasive abilities of sN1 and sN2 cells are significantly increased compared to P1 and P2 cells. However, no significant differences in proliferation activity were observed between these stable cell lines (data not shown). Simultaneously, invasive ability was dramatically increased when cells were transiently transfected with the sNEDD4 expression plasmid. Notably, cells transfected with full-length NEDD4 displayed only a slight increased in invasive ability (Figures 3B and S4). Conversely, invasive ability was decreased in sN1 cells infected with NEDD4-CDS shRNA (TRCN0000007551) expression virus, which eliminate endogenous NEDD4 and exogenous sNEDD4 simultaneously, compared to those infected with LacZ shRNA (TRCN0000231722; Figure 3C). Upon infection of sN1 cells with NEDD4–3′UTR shRNA (TRCN0000272425) expression virus, which suppresses endogenous NEDD4 only, invasive ability was maintained (Figure 3C). Invasive ability, coincident with higher sNEDD4 expression of sN1-shNEDD4–3′UTR cells, was further enhanced, compared with that of sN1-shLacZ cells. This finding may be attributed to the fact that cells expresses more sNEDD4 to compensate for the function of NEDD4 (Figure 3C).

### sNEDD4 promotes tumorigenesis and metastasis *in vivo*

**Figure 4:**
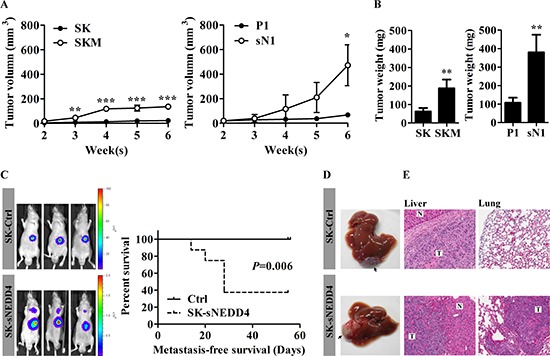
sNEDD4 promotes tumorigenesis and metastasis *in vivo* **(A)** SK, SKM, P1 and sN1 cells were subcutaneously injected into the flanks of nude mice, respectively, and tumor volumes measured once a week (*n* = 4 for each group). Tumor growth curves are shown. **(B)** Six weeks later, tumors were excised and weighed. C–E, Tumors dissected from mice subjected to subcutaneous injection with control and SK-sNEDD4 cells were introduced orthotopically into nude mice and monitored weekly with IVIS. **(C)** left, Representative IVIS images of mice 5 weeks after tumor implantation (9 mice/group). The metastasis-free survival curves are shown (C, right). Mice were sacrificed 8 weeks after implantation. Livers (the implantation site is highlighted with arrows in panel **D)** and lungs were excised, fixed and embedded in paraffin. Hematoxylin & Eosin (H&E) staining was performed on liver **(E, left)** and lung **(E, right)** tissue sections. N, normal; T, tumor; *P < 0.05; **P < 0.01; ***P < 0.001.

The subcutaneous model of human HCC in nude mice was used to assess the effects of sNEDD4 on tumorigenesis *in vivo*. SK, SKM, P1 and sN1 cells were injected subcutaneously into the flanks of male nude mice, and tumor growth curves and endpoint tumor weights obtained, as shown in Figure 4A and 4B. Tumors derived from SKM and SN1 cells grew significantly faster than those from SK and P1 cells, respectively (Figure 4A). Consistent with this, the endpoint tumor weights of SKM and sN1-derived tumors were significantly higher than those of tumors derived from SK and P1, respectively (Figure 4B). Subsequently, the metastatic abilities of control and sNEDD4-overexpressing SK cells were confirmed using an orthotopic mouse model. Metastasis was monitored with *in vivo* imaging system (IVIS) weekly after implantation of tumor mass. Lung metastatic signals were observed in three out of eight mice implantated with sNEDD4-expressing tumors 3 weeks later. More than half the mice (5/8) harbored lung metastatic signals 5 weeks later. In contrast, lung metastatic signals were not observed in mice in the control group, even at 8 weeks after implantation (Figure 4C, left). Metastasis-free survival of mouse bearing control or SK-sNEDD4 tumor was presented as Kaplan-Meier survival curves (Figure 4C, right). The implanted sNEDD4-expressing tumors grew faster than control tumors and invaded peripheral liver tissues, while a clear rim separating tumors from peripheral liver tissues was observed in control tumors (Figure 4D and 4E left panel). Lung metastases were confirmed via H&E staining of lung tissue sections (Figure 4E, right panel).

### sNEDD4 augments cell invasiveness through upregulation of MMP9 expression

**Figure 5:**
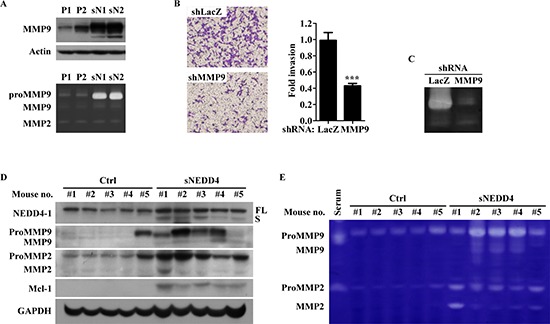
sNEDD4 promotes tumor invasion through MMP9 **(A)** MMPs protein levels and activities were determined using Western blot (upper) and gelatin zymography (lower) analyses. **(B)** Invasion abilities of sN1 cells infected with LacZ shRNA (shLacZ) or MMP9 shRNA (shMMP9) were determined with the Transwell invasion assay, and the corresponding MMP9 activities shown in panel **(C)** Western blot **(D)** and gelatin zymography **(E)** were performed with proteins extracted from primary tumors of orthotopic mouse model.

Since matrix metalloproteinase (MMP) 2 and MMP9 are significantly related to invasion, protease properties were additionally determined using Western blot and gelatin zymography. As shown in Figure 5A, MMP9, but not MMP2, protein (upper panel) and activities (lower panel) were increased in stable sNEDD4-overexpressing cells. Moreover, the invasive ability of sN1 cells was decreased upon infection of cells with MMP9 shRNA (shMMP9, TRCN0000373061) expression virus (Figure 5B and 5C). MMP9 expression levels (Figure 5D) and activities (Figure 5E) of orthotopic primary tumors were additionally determined. sNEDD4-overexpressing tumors showed higher gelatin metalloproteinase enzyme (MMP9 or MMP2) levels and activities than control tumors. These findings collectively support that activation of MMP9 serves as one of the mechanisms underlying sNEDD4 regulated tumor metastasis.

### sNEDD4 protects cells from nutrient deficiency-induced apoptosis

**Figure 6:**
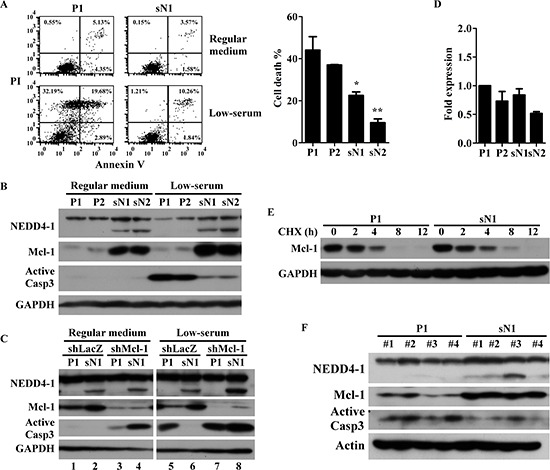
sNEDD4 upregulates Mcl-1 to overcome nutrient deficiency-induced apoptosis Stable cells were treated in the presence or absence of low-serum conditions (medium with 1% serum) for 48 h, followed by Annexin V-propidium iodide (PI) double staining, and the proportion of apoptotic cells determined via flow cytometry **(A)** Protein expression patterns of these cells were determined using Western blot **(B) (C)** P1 and sN1 cells were transiently transfected with control (shLacZ) or Mcl-1 shRNA (shMcl-1) for 48 h, cultured in low-serum conditions for a further 48 h, and Casp3 activation analyzed. **(D)** qRT-PCR was used to determine Mcl-1 mRNA levels of stable cells. **(E)** Mcl-1 protein stability was analyzed with the protein degradation assay. **(F)** Protein levels of sNEDD4, Mcl-1, and active Casp3 of xenograft tumors on the flanks of mice were determined. CHX, cycloheximide; *P < 0.05; **P < 0.01.

Since nutrient deprivation conditions causing cancer cell apoptosis are common to the tumor microenvironment, tolerance for nutrient deficiency in control and sNEDD4-overexpressing cells was determined. After 48 h incubation in low-serum conditions, cells were harvested and apoptosis detected with the FITC Annexin V Apoptosis Detection Kit (BD Pharmingen). As shown in Figure 6A, the proportion of apoptotic cells was significantly lower in sN1 and sN2 cells, compared to P1 and P2 cells. In addition, active caspase 3 (Casp3) was induced significantly in P1 and P2 cells, but only slightly in sN1 and sN2 cells, under low-serum conditions (Figure 6B). Bcl-2 family proteins are known critical regulators of apoptosis. Therefore, expression levels of several Bcl-2 proteins in stable cells were assessed. Our data showed that myeloid cell leukemia 1 (Mcl-1), one of the anti-apoptosis Bcl-2 family proteins, is significantly upregulated in sN1 and sN2 cells, compared to P1 and P2 cells (Figure 6B). In contrast, we observed no significant differences in Bcl-2, Bim and Bid levels among these stable cell lines (data not shown). Knockdown of Mcl-1 abrogated the protective effect of sNEDD4 against apoptosis (Figure 6C, lane 6 and 8). No significant differences in Mcl-1 mRNA levels were evident among the cell lines examined (Figure 6D). The protein stability of Mcl-1 was further determined. As shown in Figure 6E, Mcl-1 was detectable in sN1, but not P1 cells, after 8 h treatment with cycloheximide (CHX), indicating that Mcl-1 is more stable in sN1 than P1 cells. sNEDD4, Mcl-1 and active Casp3 levels in tumors excised from flanks of nude mice were further analyzed (Figure 6F). sNEDD4 was specifically detected in sN1-derived tumors. Mcl-1 was upregulated, and conversely, active Casp3 was downregulated in sN1-derived tumors. This result was consistent with data obtained *in vitro* (Figure 6B). Inhibiting of apoptosis by sNEDD4 may contribute to tumorigenesis in mice.

### E3 activity is required for impacts of sNEDD4 on cell invasiveness

**Figure 7:**
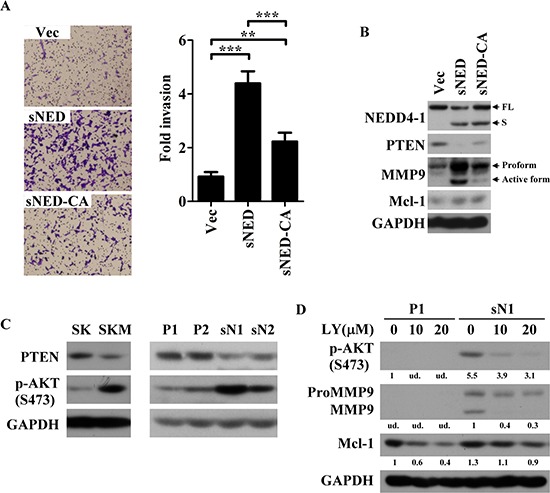
E3 activity is critical for sNEDD4 function **(A)** Transwell invasion assay was performed to analyze the invasive ability of stable cells. Left, images of traversed cells staining with crystal violet; Right, quantitative result of Transwell invasion assay; **(B–D)** Protein expression patterns of cells were determined using Western blot. D, P1 and sN1 cells were treated with LY294002 at 10–20 μM for 24 h prior harvested. The quantitative results were shown under the images. Vec, vector control stable cells; sNED, sNEDD4-overexpressing stable cells; sNED-CA, sNEDD4-CA-overexpressing stable cells; **P < 0.01; ***P < 0.001; LY, LY294002; S473, serine 473; ud., undetectable.

To unravel whether the E3 activity of sNEDD4 is required for its function on tumor invasion, a sNEDD4 mutant was made by replacing three cysteine residues (critical to its E3 activity [5]) with alanine (sNEDD4-CA) on its HECT domain. Subsequently, sNEDD4-CA-overeexpressing stable cells were used to determine the effect of invasion ability by Transwell assay. As shown in Figure 7A, the invasive ability of SK-sNEDD4-CA cells was significantly decreasing, comparing to sNEDD4-overexpressing cells. This data reveals that E3 activity is required for sNEDD4 to promote tumor invasion. PTEN, one of the target ubiquitinated by NEDD4, expression level of stable cells was determined using Western blot. A dramatically or moderately decreasing of PTEN was observed in sNEDD4-overexpressing or NEDD4-overexpressing stable cells, respectively comparing to the control cells (Supplementary Figure S4). By comparing to sNEDD4-overexpressing cells, restoration of PTEN expression was observed in sNEDD4-CA-overexpressing cells (Figure 7B). These results indicate that sNEDD4 may have stronger E3 activity, responsible for PTEN degradation, than NEDD4.

### PTEN-PI3K-AKT axis is possible mechanism underlying sNEDD4 functions

PTEN inhibits PI3K-AKT signaling pathway. Phosphorylated-AKT (p-AKT) expression level was determined by Western blot analysis. As shown in Figure 7C, p-AKT increased alone with PTEN decreasing upon endogenously (SKM) and ectopically (sN1 and sN2) overexpressing of sNEDD4. MMP9 and Mcl-1 were decreased while treated sN1 cell with selective PI3K inhibitor, LY294002 (Figure 7D). The data reveals that PTEN-PI3K-AKT axis is one of the potential mechanisms underlying sNEDD4 functions.

## DISCUSSION

In our study, a transplantation experimental metastasis model was selected to develop cells lines with progressively higher metastatic ability, as described by Fidler *et al*. (1977). Using this *in vivo* selection model, the group originally generated B16-F10 melanoma cells and confirmed their high metastatic potential [6–8]. Here, we developed potent metastatic SKM cells in a similar manner, and subsequently performed genomic analyses to identify metastasis-associated genes. Although species-specific incompatibility and manipulation in immunocompromised mouse, which eliminates the involvement of the immune system in tumor progression, are yet to be resolved, transplantation xenograft models remain the most prevalent model type to study human tumor metastasis. Edwin *et al*. [9] showed that RhoC is overexpressed and essential for tumor metastasis of selected highly metastatic melanoma cells. Associations of RhoC expression with lymphatic metastases and poor survival has been confirmed in melanoma [10]. Clearly, genomic analysis of potent metastatic cells selected with an *in vivo* model provides a useful tool for metastasis studies.

Another drawback of the experimental metastasis model is that only post-intravasation steps are modeled. Post-intravasation steps specifically assigned to metastasis colonization are thought to be the most inefficient step of metastasis [11]. Using quantitative cell-fate analysis, Luzzi *et al*. [12] demonstrated that over 87% of the injected cells are present in the liver 90 min after injection, and 83% undergo extravasation and arrest in the liver. However only 2% of the injected cells formed micrometastases and 0.02% displayed progressive metastases. The timing and steps of extravasation were identical for cells with different metastatic abilities and transformation status, while metastasis colonization behaviors reflected the differences in these cells [13, 14]. For clinical therapy, metastatic colonization is suggested the most appropriate intervention time-point [15]. The option of preventing occurrence of metastasis appears impractical, since disseminated cells may already be present while the primary tumor is diagnosed. Therefore, targeting the inefficient biological process may be a more effective, easy and economical method of therapy. Accordingly, identification of molecules and genes contributing to colonization via the experimental metastasis assay should aid in clarifying the mechanisms underlying the rate-limiting step of metastasis and therapeutic development. Although SKM cells were selected using the experimental model, the metastatic ability of SK-sNEDD4 cells was investigated using a spontaneous assay comprising the entire steps of metastasis. We have demonstrated that SK-sNEDD4 cell-derived tumors exhibited metastasis to the lung more effectively than control cells.

In the current study, we identified a novel transcript of NEDD4, sNEDD4, which is overexpressed in potent metastatic SKM cells. Based on a website tool to predict alternative translational start sites and subsequent verification with qRT-PCR, we proposed that sNEDD4 is translated from A^633^TG into a short form lacking the N-terminal C2-domain. The C2-domain serves as auto-inhibition machinery by interacting with the C-terminal HECT-domain of NEDD4 [16]. Accordingly sNEDD4 may resemble the constitutively active form of NEDD4. Our experiments showed that overexpression of sNEDD4 increases SK cell invasive ability more significantly than transfection with FL-NEDD4 (Figure 3B and S4). The identification of upstream pathways controlling sNEDD4 expression may aid in uncovering the mechanisms underlying HCC progression.

Our experiments demonstrated increased MMP9 expression and activity upon induction of sNEDD4 in SK, which is crucial for the invasive ability of SK-sNEDD4 cells, as confirmed with the Transwell invasion assay. As expected, MMP9 serves to break down the extracellular matrix to facilitate tumor invasion. Roles of MMPs in growth and angiogenesis of primary and secondary tumor, other than their protease function, have been established. The reduced metastatic ability of tissue inhibitors of metalloproteinase (TIMP)-1 overexpressing melanoma cells is due to micrometastatic colony formation, but not defective extravasation [17, 18]. Hiratsuka *et al*. [19] reported that tumor-associated macrophage-induced-MMP9 expression optimizes the microenvironment required for secondary malignant cell growth. In addition to MMP9, MMP2 expression and activity were increased in all SK-sNEDD4 tumors of the orthotopic model. The involvement of MMP2 in metastasis colonization has been previously confirmed [20, 21]. However, elevated MMP2 expression and activity were not observed in SKM and SK-sNEDD4 cell cultures. In view of these findings, we speculate that specific factors from the tumor microenvironment are critical for MMP2 activation by sNEDD4. Accordingly, MMP2 and MMP9 may function in primary tumor growth, invasion, and colonization, facilitating SK-sNEDD4 cell metastasis to the lung.

We have presented evidence that sNEDD4 stabilizes Mcl-1 to alleviate nutrient deficiency-induced apoptosis. The reason why E3 ubiquitin ligase does not promote, and instead protects protein from degradation remains to be established. Mcl-1 is a short half-life protein with a quick turnover though the proteasome degradation pathway. The F-box and WD repeat domain-containing 7, Fbw7, is the E3 ligase responsible for Mcl-1 degradation [22]. Substrates of Fbw7 have been extensively characterized, however, little is known about the upstream regulation mechanisms of Fbw7. Kimura *et al*. [23] demonstrated that Fbw7 is a transcriptional target of p53. Xu and co-workers reported that NEDD4 ubiquitinates and stabilizes Mdm2 to inhibit the p53 response [24]. We speculate that regulation of the Mdm2-p53-Fbw7 axis by sNEDD4 is a possible mechanism underlying stabilization of the Mcl-1 protein.

Protein level of MMP9 and Mcl-1 in human HCC specimens was determined using Western blot (Supplementary Figure S5). Correlations of sNEDD4 with MMP9 or Mcl-1 level were analyzed. T/N ratios of Mcl-1 were significantly correlated with that of sNEDD4 (Spearman's correlation; *P* = 0.043; correlation coefficien *t* = 0.266), but not MMP9. The discrepancy may be due to a small sample size, since MMP9 was detectable in only 28 out of 70 paired specimens. sNEDD4 may regulate Mcl-1 and MMP9 to augment tumor invasion in a subgroup of patients.

Analysis of the clinical significance of sNEDD4 revealed that sNEDD4 is a poor prognostic factor for HCC in males, but not females. These results imply that sNEDD4 has an impact on gender-related molecules, such as the androgen receptor (AR), during HCC progression. Pathologic roles of AR in HCC have been confirmed [25], but conflicting results on prognosis for HCC reported to date [26–28]. Interestingly, NEDD4 has been shown to negatively regulate the stability of AR in prostate cancer cells [29]. Thus, investigation of the interactions between NEDD4 and AR in HCC is warranted in the future.

In conclusion, sNEDD4 is a novel metastasis-associated gene, which regulates tumor invasion, apoptosis, and even colonization to augment cancer metastasis. The prognostic potential of sNEDD4 was confirmed. Further study focousing on the mechanisms underlying sNEDD4-regulated tumor progression are critical to elucidate the processes involved in metastasis and provide an opportunity to improve prognosis and identify new therapeutic target for HCC.

## METHODS

### Cell line and cell culture

The human HCC cell line, SK-Hep-1 (SK; HTB-52), was obtained from American Type Culture Collection (ATCC) and cultured as described previously [30]. The cell line was authenticated by assessments of short tandem repeat loci following database comparison. To generate potent metastatic cell lines, SK cells were selected using the Transwell invasion assay (see below). Traversed cells were harvested and cultured to obtain SK-T (T represents “Transwell”) cells. Subsequently, SKT cells (1×10^7^ cells in 150 μl PBS) were introduced intravenously into severe combined immunodeficiency (SCID) mice. Six weeks later, lung metastatic foci were harvested, chopped, trypsinized, and cultured in Dulbecco's Modified Eagle Medium. Cells were passaged 3–4 times to generate pure liver cancer cells, designated SK-M (M represents “Metastasis”) cells.

### DNA microarray

cDNA microarray and Affymetrix oligomicroarray were performed as described previously [31, 32]. The gene list obtained upon comparison of SK and SKM cells was filtered based on fold changes greater than 1.5. Results obtained from the cDNA microarray were crossed with those from the Affymatrix oligomicroarray. Genes identified simultaneously in both cDNA and Affymetrix microarrays were selected for further study.

### qRT-PCR

Total RNA was extracted and cDNA strands synthesized using the Superscript III kit for RT-PCR (Life Technologies, USA). Real-time quantitative RT-PCR was performed using SYBR green, and detected using the ABI PRISM 7500 system (Applied Biosystems, UK).

### Immunoprecipitation and LC-MS/MS

Cells were harvested and washed with cold PBS three times. PBS plus 0.1% Nonidet P-40 (NP-40; Sigma-Aldrich) was used to lyse cells at 4°C for 30 min. Cell debris were removed by centrifugation at 12000 rpm for 10 min. The NEDD4 primary antibody (5 μl for 500 μg proteins) was used to immunoprecipitate NEDD4 from cell lysates. Protein A/G Plus-Agarose (Santa Cruz) was used to pull-down the immunoreactive complex, as recommended by the manufacturer. Finally, agarose beads were re-suspended in sample buffer and subjected to SDS-PAGE, followed by silver staining. Bands of interest were excised from the gel and subjected to in-gel tryptic digestion and LC-MS/MS analysis, as described previously [33].

### Study population

Seventy primary paired HCC tissues and associated clinical information were provided by the Taiwan Liver Cancer Network (TLCN) funded by the National Science Council. This study was approved by our Institutional Review Board and the TLCN User Committee. Sixty-one patients subjected to primary definitive surgery without pretreatment adjuvant therapy were enrolled. The median follow-up time of surviving patients among these cases was 4.7 years (range, 0.7–7.7 years). Overall, 32 patients with relapse and 21 deaths were recorded during the follow-up period. Patient and tumor characteristics are listed in Supplementary Table S2.

### Xenograft mouse model

Cells were injected subcutaneously into the flanks of nude mice (BALB/cAnN.Cg-Foxn1^nu^/CrlNarl) at concentration of 6×10^6^/200 μl. Tumor mass was monitored and measured weekly with calipers. Mice were sacrificed 6 weeks later, and the endpoint tumor weights recorded.

### Orthotopic mouse model

Tissues of subcutaneously grown HCC cells were cut into small pieces about 1mm^3^ in size. Tumor pieces were implanted into livers of nude mice as described earlier [34]. Growth and metastasis of implanted tumors were monitored with an IVIS once a week [35], and mice were sacrificed 8 weeks later. Livers and lungs were excised and stored at −80°C or fixed and embedded in paraffin. Primary Liver tumors and metastatic lung foci were confirmed via H&E staining.

### Transwell invasion assay

Cells were trypsinized, washed once with PBS and re-suspended in serum-free medium. Cells were loaded on to the upper chamber (Corning-Costar 3494 Transwell, Lowell, MA, USA; 5×10^4^ cells in 150 μl) pre-coated with Matrigel (Becton, Dickinson and Company, Franklin Lakes, NJ) for 2 h at 37°C. Medium supplemented with 20% fetal bovine serum (FBS) was added to the lower chamber. After a 24 h incubation period, traversed cells were fixed and stained with crystal violet. Cell numbers per field were counted.

### Gelatin zymography

Supernatant fractions of cells cultured for 24 h were collected and concentrated using Amicon Ultra-4 Centrifugal Filter Devices (Merck Millipore Ltd.). Equal amounts of proteins were separated via 8% SDS-PAGE with 0.1% gelatin (Sigma-Aldrich). The gel was incubated in reaction buffer (40 mM Tris-HCl, pH 8, 10 mM CaCl_2_, and 1% NaN_3_) at 37°C overnight, stained with 0.25% Coomassie Brilliant Blue R-250 in 10% acetic acid and 50% methanol for 30 min, and de-stained with 10% acetic acid and 20% methanol twice for 30 min.

### Flow cytometry detection of apoptotic cells

Cells were cultured in regular or low-serum medium (1% FBS) for 48 h and harvested, followed by washing twice with PBS. Annexin V and propidium iodide (PI) double staining was performed with the FITC Annexin V Apoptosis Detection Kit (BD Biosciences), as recommended by the manufacturer. The percentage of apoptotic cells was detected using the FACScan flow cytometer (Becton Dickinson, San Jose, CA) running CellQuest software.

### Protein degradation assay

Cells were pre-treated with MG132 (10 μM; Sigma-Aldrich, St. Louis, MO) for 4 h. The medium was refreshed and CHX (10 ng/ml; Sigma-Aldrich) added to block new protein synthesis. Cells were harvested at the indicated time-points after treatment with CHX.

### Statistical analysis

Kruskal-Wallis *H* test and Mann-Whitney test were applied to compare mean data from groups. Survival of patients with death (overall survival, OS) or relapse (recurrence-free survival, RFS) as events was analyzed using the log-rank test. Kaplan-Meier survival curves are shown. *P* values < 0.05 were considered significant. SPSS statistical software was used for statistical analyses.

## SUPPLEMENTARY FIGURES AND TABLES


