# Supplementary material for: A novel small-form NEDD4 regulates cell invasiveness and apoptosis to promote tumor metastasis
Source: Oncotarget. 2015 Mar 20;6(11):9341–54. doi: 10.18632/oncotarget.3322 (PMC4496221; doi:10.18632/oncotarget.3322)
Supplement: Supplementary file 1 [file oncotarget-06-9341-s001.pdf]

SUPPLEMENTARY FIGURES AND TABLES

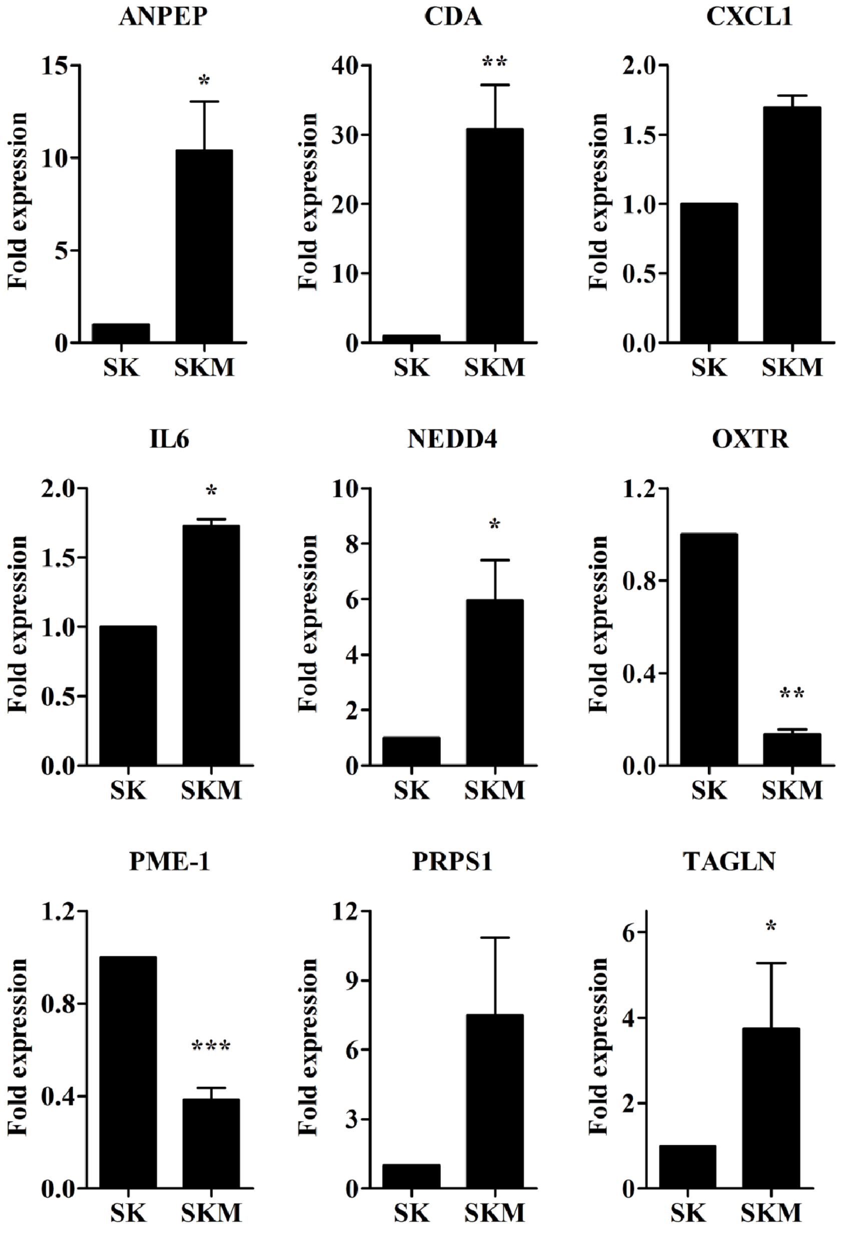

Supplementary Figure S1: Verify the results of DNA microarray analyses The mRNA level of metastasis-related genes was verified using qRT-PCR.

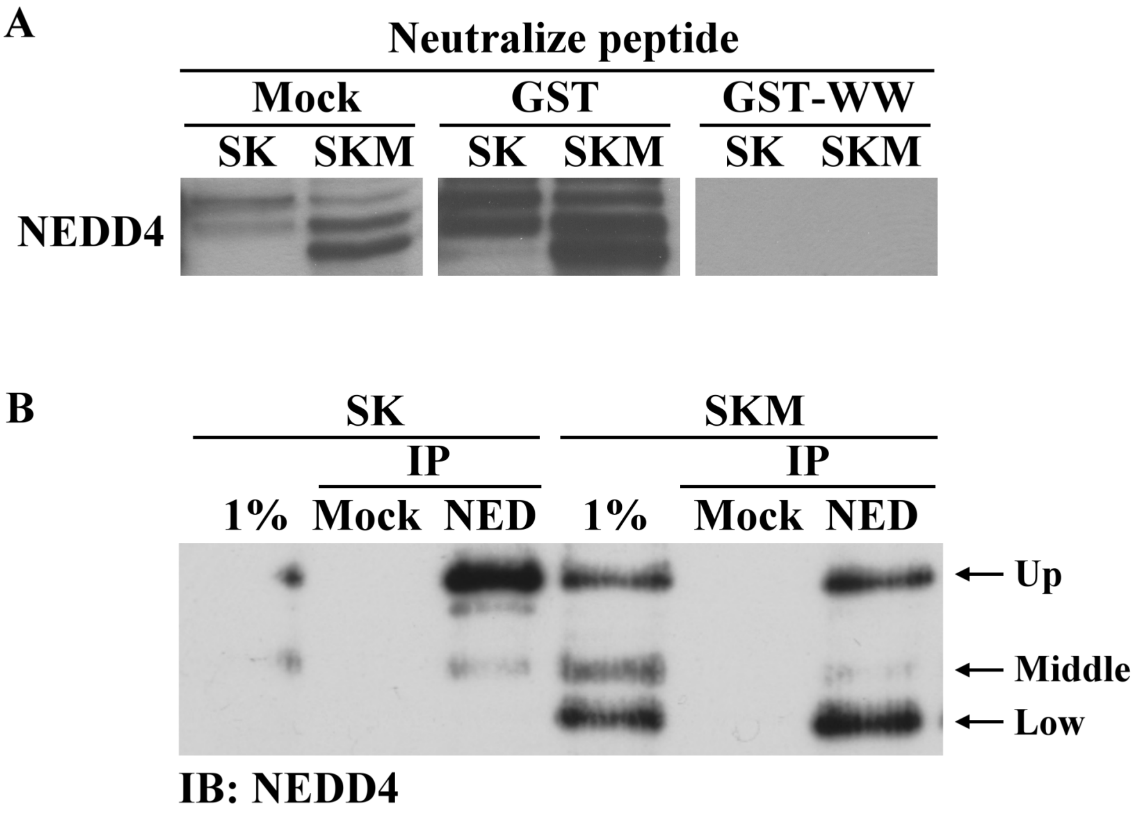

Supplementary Figure S2: NEDD4 antibody specificity (A) Antibody specificity was tested using Western blot with control (GST) or NEDD4-neutralizing peptide (GST-WW). **(B)** Cell lysates of SK or SKM cells were immunoprecipitated with the NEDD4 antibody (NED) and separated via SDS-PAGE, and the signals visualized via Western blot. GST, glutathione-S transferase; GST-WW, GST fusion NEDD4-WW domain; IP, immunoprecipitation; 1%, 1% in-put.

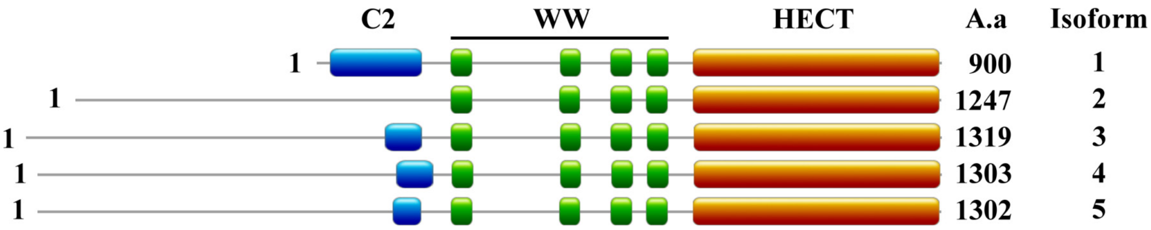

Supplementary Figure S3: Schematic diagram of NEDD4 protein structure The schematic cartoon represents protein structures of five NEDD4 isoforms.

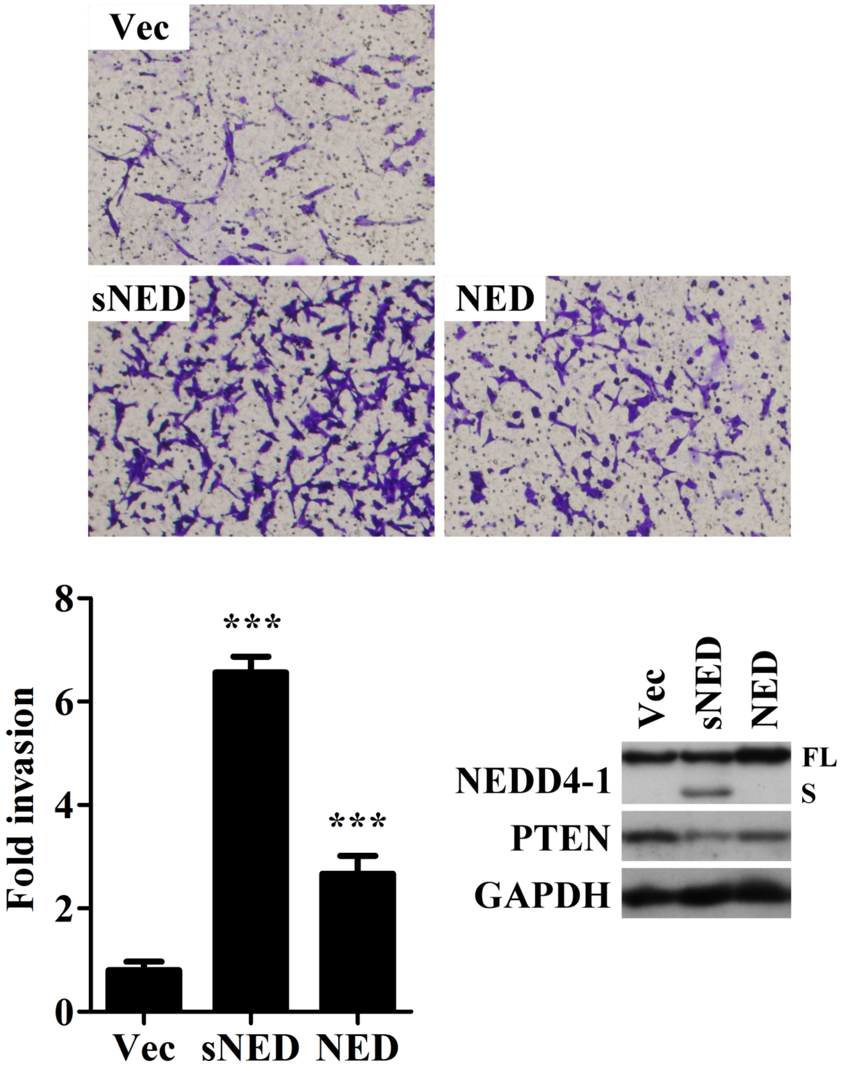

Supplementary Figure S4: sNEDD4 promotes tumor cell invasion *in vitro* The invasive ability of control (Vec), sNEDD4-overexpressing (sNED) and NEDD4-overexpressing (NED) stable cell lines was determined with the Transwell invasion assay. NEDD4 and sNEDD4 protein levels of each cell were determined using Western blot. ***$P < 0.001$.

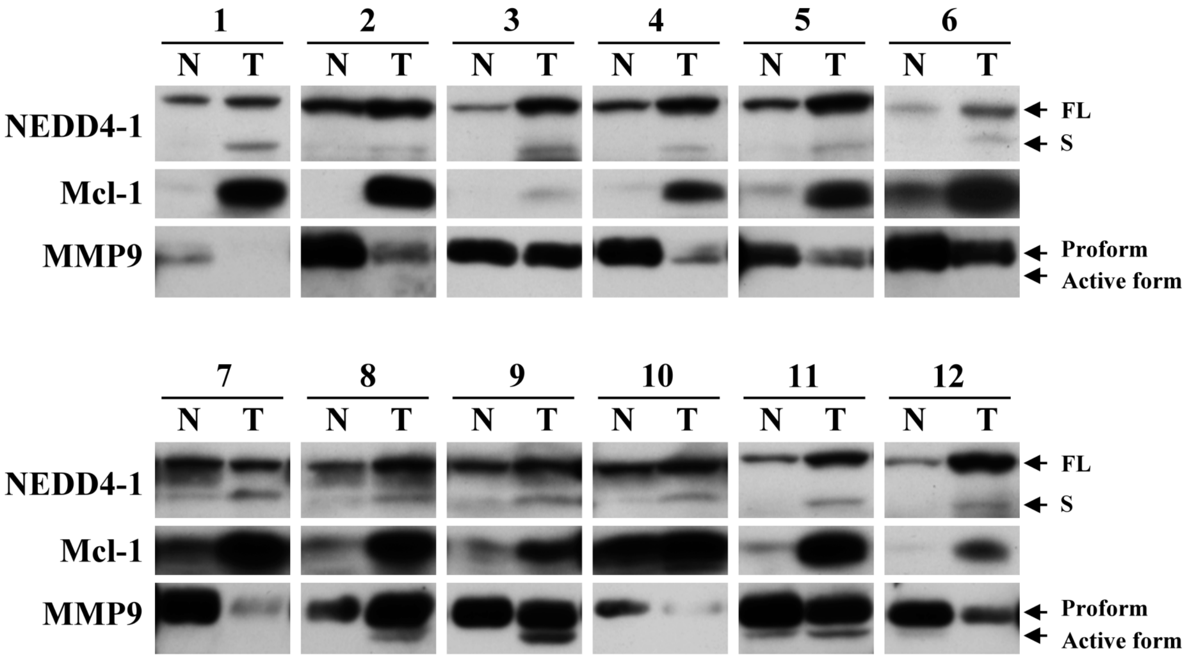

Supplementary Figure S5: Expression of MMP9 and Mcl-1 in HCC Protein levels of MMP9 and Mcl-1 of 70 paired HCC specimens were determined using Western blot. Represented results were shown. N, adjacent normal tissue; T, tumor tissue; FL, full-length NEDD4; S, sNEDD4.

Supplementary Table S1: Peptide representing NEDD4 and NEDD4-L identified by LC-MS/MS

Immunoreactive band	Gene name	Corresponding peptides
Upper	NEDD4	VRVIAGIGLAK
		DILGASDPYBRBTLYD
		PMNGVLTSVQ
		LLFEVFDENR
		DFVLHPR
		TYYVNHESR
		LTIFGNSAVSQPASSSNHSSR
		IFYINHNIK
		LENVAITGPAVPYSR
		RATVLEDSYRR
		GLDYGGVAR
		VAGMAVYHGK
		NGGSEIVVTNK
		AVLMMDSEK
		LLQFVTHTSR
Middle	NEDD4	GLDYGGVAR
		LLQFVTGTSR
	NEDD4L	QINQEAAHR
		TSPQELSEELSR
		SLSSPTVTLSAPLEGAK
		DTLSNPQSPQPSYPYNSPKPQHK
		FPVHMR
		TFYIDHNSK
		LQNPAITGPAVPYSR
		KPADIPNRFEMK
		NNIFEESYRR
		GLDYGGVAR
		VAGLAVFHGK
		AVLLMDAEKR
		LLQFVTGTSR
Lower	NEDD4	IFYINHNIK
		LENVAITGPAVPYSR
		FIIDEELFGQT
		LLQFVTGTSR

Supplementary Table S2: Patient and tumor characteristics

Parameter	*n* (%)
Median age (range; years)	60 (23–85)
Gender	
Male	39 (63.9%)
Female	22 (36.1%)
Stage	
I	27 (44.3%)
II	20 (32.8%)
III	14 (22.9%)
Histological type	
Solitary	44 (72.1%)
Multiple	17 (27.9%)
Grading	
1	3 (4.9%)
2	43 (70.5%)
3	15 (24.6%)
Tumor size	
≦ 4cm	31 (50.8%)
> 4cm	30 (49.2%)
Vascular invasion	
Absence	30 (49.2%)
Capsular vein	2 (3.3%)
Portal vein	29 (47.5%)
Cirrhosis	
No	38 (62.3%)
Yes	23 (37.7%)
Viral status	
HBV or HCV	56 (91.8%)
NBNC#	5 (8.2%)

#patients negative for both HBV and HCV
